# Draft genome of hydrocarbon-degrader *Burkholderia cepacia* BCTT, isolated from soil chronically polluted with crude oil in Trinidad

**DOI:** 10.1128/mra.00902-24

**Published:** 2024-10-29

**Authors:** Amanda Christine Ramdass, Sephra Nalini Rampersad

**Affiliations:** 1 Biochemistry Research Lab (Rm216), Department of Life Sciences, Faculty of Science and Technology, The University of the West Indies, St. Augustine, Trinidad and Tobago; Rochester Institute of Technology, Rochester, New York, USA

**Keywords:** bioremediation, *Burkholderia*, polyaromatic hydrocarbon, biocatalyst

## Abstract

*Burkholderia cepacia* BCTT*,* isolated from chronically polluted soil in Trinidad, shows a capacity to survive in crude oil as a sole carbon source. Here, we report its high-quality draft genome sequence and highlight those pathways and genes involved in xenobiotic degradation. These data give a clearer insight into this organism’s biotechnological potential.

## ANNOUNCEMENT

*Burkholderia cepacia* is a Gram-negative bacterium belonging to the *B. cepacia* complex (Bcc) (1). Members of this complex occupy a range of different environments, and some are opportunistic pathogens (2, 3). Bcc bacteria harbor large genomes and demonstrate extraordinary degradative abilities, metabolic versatility, and engage multiple strategies for niche adaptation (4). One example of metabolic diversity is the ability to metabolize aromatics, nitroaromatics, and halogenated compounds (1, 5). While indigenous strains of *B. cepacia* are associated with degrading crude oil and polyaromatic hydrocarbons (PAHs) (6, 7), there are no available reports on the whole-genome sequence of a hydrocarbonoclastic strain.

*B. cepacia* BCTT was isolated from soil chronically polluted with crude oil within 0.5 m to an abandoned oil well in Trinidad in 2017. This strain was previously isolated on Reasoner’s 2A agar (R2A) and identified via 16S rRNA sequencing (GenBank: MW633287 ), shown to utilize crude oil as its sole carbon source and produce extracellular lipase (8). To better determine the biodegradative versatility of this strain, we performed whole-genome sequencing.

Genomic DNA was prepared from an axenic colony grown on R2A agar at 25°C for 48 hours. DNA was extracted using a Maxwell 16 Cell DNA Purification kit following the manufacturer’s instructions. DNA quality and concentration were determined by agarose gel electrophoresis analysis and spectrophotometry (Qubit 3.0 fluorometer). Novogene Corporation Inc. (Sacramento, CA) sequenced the libraries on an Illumina HiSeq platform using paired-end 150-bp reads. A library was prepared using the NEBNext DNA Library Prep Kit, following the instructions strictly. To check the prepared library, a Qubit 2.0 fluorometer was first used to determine the concentration, an Agilent 2100 bioanalyzer to assess insert size, and finally quantitative real-time PCR to detect the effective concentration (> 2 nM). A total of 4,072,980 raw reads were generated (Q20% of 94.44; Q30% of 86.14) and assembled in Shovill using the Spades assembler (v1.1.0) (9) and validated using QUAST (v5.0.2). The average sequencing depth was 129.11X. A BUSCO (v5.5.0) score of 99.8% indicated high completeness of the assembly (10).

Expert annotation of the genome was completed by the Joint Genome Institute (JGI) Integrated Microbial Genomes Expert Review (IMG/ER) annotation system (IMG (IMGAP v5.1.5) Gene calling program: GeneMark.hmm-2 v1.25_lic; https://img.jgi.doe.gov/cgi-bin/mer/main.cgi). The resulting high-quality draft genome consists of 8,358,227 bp with an N_50_ value of 4,561,718 bp, G + C content of 66.81%, 7,656 protein-coding sequences, and 17 rRNA and 70 tRNA genes (Table 1).

**TABLE 1:** Annotation statistics of *B. cepacia* BCTT

	Number	% of total
DNA, total number of bases	8,358,227	100.00%
DNA coding number of bases	7,339,435	87.81%
DNA G + C number of bases	5,584,526	66.81%^*a*^
DNA scaffolds	81	100.00%
Gene total number	7,786	100.00%
Protein-coding genes	7,656	98.33%
Regulatory and miscellaneous features	25	0.32%^*b*^
RNA genes	91	1.17%
rRNA genes	17	0.22%
5S rRNA	3	0.04%
16S rRNA	3	0.04%
18S rRNA	1	0.01%
23S rRNA	9	0.12%
28S rRNA	1	0.01%
tRNA genes	70	0.90%
Other RNA genes	4	0.05%
Protein-coding genes with function prediction	6,201	79.64%
Without function prediction	1,455	18.69%
Protein-coding genes with enzymes	2,008	25.79%
Protein-coding genes connected to KEGG pathways	2,378	30.54%

^*a*^ GC percentage shown as count of Gs and Cs divided by the total number of bases. The total number of bases is not necessarily synonymous with the total number of Gs, Cs, As, and Ts.

^*b*^ Regulatory or miscellaneous genes are genes that are not classified as CDS, a type of RNA, or a pseudogene, but as “unknown” or “other” by the source provider.

**Fig 1:**
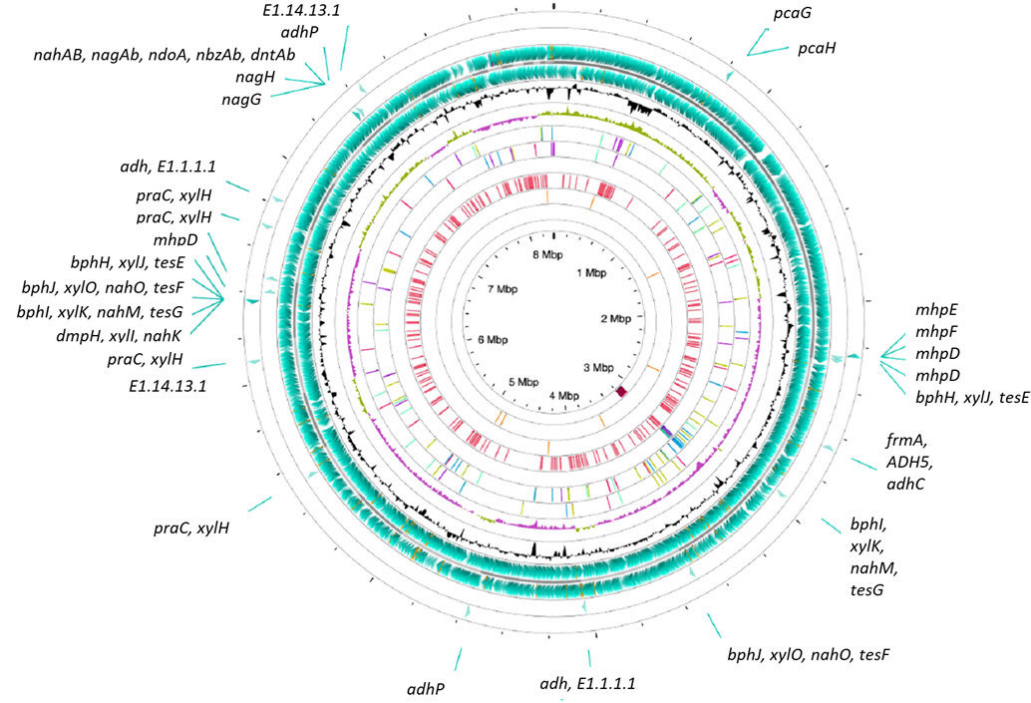
Circular map of the draft genome of *B. cepacia* BCTT. The map was generated through Proksee (https://proksee.ca/). Starting from the outermost ring: ring 1: PAH genes (+), ring 2: PAH genes (-), ring 3: annotated genes (+), backbone (contigs), ring 5: annotated genes (-), ring 6: GC content, ring 7: GC skew, ring 8: mobile genetic elements (+) (mobileOG-db v1.1.3), ring 9: mobile genetic elements (-) (mobileOG-db v1.1.3), ring 10: prophage regions (Phigaro v1.0.1), ring 11: putative horizontal gene transfer events (Alien Hunter v1.1.0), ring 12: antibiotic resistance genes (+) (CARD RGI v1.2.1), ring 13: antibiotic resistance genes (-) (CARD RGI v1.2.1), and ring 14: dsDNA and ssDNA virus genomes (phage) (VirSorter v1.1.1). Genes detected in pathways for PAH degradation included the following: fluorene, anthracene, benzo[a]pyrene, naphthalene - naphthalene 1,2-dioxygenase ferredoxin component (*nahAb, nagAb, ndoA, nbzAb,* and *dntAb*); anthracene, naphthalene, phenanthrene, dioxin (dibenzo-p-dioxin and dibenzofuran): salicylate hydroxylase (EC:1.14.13.1); PAH: protocatechuate 3,4-dioxygenase, alpha subunit (*pcaG*), protocatechuate 3,4-dioxygenase, beta subunit (*pcaH*); naphthalene: alcohol dehydrogenase (*adh*, E1.1.1.1), alcohol dehydrogenase, propanol-preferring (*adhP*), S-(hydroxymethyl)glutathione dehydrogenase / alcohol dehydrogenase (*frmA, ADH5,* and *adhC*), salicylate 5-hydroxylase large subunit (*nagG*), salicylate 5-hydroxylase small subunit (*nagH*); dioxin (dibenzo-p-dioxin and dibenzofuran): 2-keto-4-pentenoate hydratase (*mhpD*), 4-hydroxy 2-oxovalerate aldolase (*mhpE*), acetaldehyde dehydrogenase (*mhpF*), 2-oxo-3-hexenedioate decarboxylase (*dmpH, xylI,* and *nahK*), two oxopent-4-enoate/cis-2-oxohex-4-enoate hydratase (*bphH, xylJ,* and *tesE*), 4-hydroxy-2 oxovalerate/4-hydroxy-2-oxohexanoate aldolase (*bphI, xylK, nahM,* and *tesG*), four oxalocrotonate tautomerase (*praC* and *xylH*), acetaldehyde/propanal dehydrogenase (*bphJ, xylQ, nahO,* and *tesF*). mobileOG-db (rings 8 and 9), identified 169 features: 27 integration/excision, 46 replication/recombination/repair, 43 phage, and 29 stability/transfer/defense 24 transfer. Phigaro (ring 10) detected one prophage region of 38.4 Kb in contig 04, of which one region is intact, 0 regions are incomplete, and 0 regions are questionable, and its most common phage was PHAGE_Escher_vB_EcoM_ECOO78_NC_041926(10). CARD (rings 12 and 13) prediction includes the following classes of antibiotics: fluoroquinolone antibiotics: tetracycline (*adeF*) and aminoglycoside (*ceoB*), glycopeptide antibiotic: *vanH* gene in the *vanO* cluster, aminoglycoside antibiotics: *amrB* and *amrA*; disinfecting agents and antiseptics (*qacG*).

Important genes associated with xenobiotic degradation (Fig. 1) were identified for BCTT, including degradation of benzoate, amino- and fluoro-benzoate, chloro-alkane/alkene, dioxin, naphthalene, nitrotoluene, PAHs, styrene, and BTEX (benzene, toluene, ethylbenzene, and xylene) (11). Reaction initiators, catechol 1,2-dioxygenase and catechol 2,3-dioxygenase, are present for aromatic ring opening during initial PAH degradation (12). Also annotated are protocatechuate degradation genes (*pcaGHIJDCBQRK*) (13) and genes encoding homogentisate 1,2-dioxygenase (14). The genome encodes biocatalysts including extracellular chitinase (15), dienelactone hydrolase (16), triacylglycerol lipases (17), and biosurfactants, e.g., surfactin and related phospholipids (18, 19), which may be industrially relevant. Genes associated with bioplastic degradative depolymerase enzymes were also identified (20). In conclusion, the genomic features of *B. cepacia* BCTT provide a better understanding of the hydrocarbonoclastic potential of this strain, especially with respect to bioremediation.

## Data Availability

This Whole-Genome Sequencing project has been deposited at DDBJ/ENA/GenBank under the accession JBGKEA000000000, and the raw reads were deposited in NCBI’s SRA under the accession PRJNA1148172. The annotation described in this paper is under the JGI GOLD Project ID Gp0618334, and annotation results can be viewed via the link https://img.jgi.doe.gov/cgi-bin/mer/main.cgi?section=TaxonDetail&page=taxonDetail&taxon_oid=2953109840 upon opening a free account with JGI.
